# Imaging of thyroid tumor angiogenesis with microbubbles targeted to vascular endothelial growth factor receptor type 2 in mice

**DOI:** 10.1186/1471-2342-13-31

**Published:** 2013-09-12

**Authors:** Marcello Mancini, Adelaide Greco, Giuliana Salvatore, Raffaele Liuzzi, Gennaro Di Maro, Emilia Vergara, Gennaro Chiappetta, Rosa Pasquinelli, Arturo Brunetti, Marco Salvatore

**Affiliations:** 1 Institute of Biostructure and Bioimaging, Italian National Research Council (CNR), Naples, Italy; 2 SDN Foundation IRCCS, Naples, Italy; 3 Dipartimento di Scienze Biomediche Avanzate, Università degli Studi di Napoli “Federico II”, Naples, 80131, Italy; 4 Dipartimento di Studi delle Istituzioni e dei Sistemi Territoriali, Università degli Studi di Napoli “Parthenope”, Naples, Italy; 5 CEINGE-Biotecnologie Avanzate s.c.a.\r.l., Naples, Italy; 6 Dipartimento di Biologia e Patologia Cellulare e Molecolare, Università degli Studi di Napoli “Federico II”, Naples, 80131, Italy; 7 Functional Genomic Unit, Istituto Nazionale Tumori G. Pascale, Naples, Italy

**Keywords:** Thyroid, Transgenic, High resolution ultrasound, Cancer, Contrast agent

## Abstract

**Background:**

To evaluate whether Contrast Enhanced Ultrasund (CEUS) with microbubbles (MBs) targeted to VEGFR-2 is able to characterize *in vivo* the VEGFR-2 expression in the tumor vasculature of a mouse model of thyroid cancer (Tg-TRK-T1).

**Methods:**

Animal protocol was approved by Institutional committee on Laboratory Animal Care. Contrast-enhanced ultrasound imaging with MBs targeted with an anti-VEGFR-2 monoclonal antibody (UCA_VEGFR-2_) and isotype control antibody (UCA_IgG_) was performed in 7 mice with thyroid carcinoma, 5 mice with hyperplasia or benign thyroid nodules and 4 mice with normal thyroid. After ultrasonography, the tumor samples were harvested for histological examination and VEGFR-2 expression was tested by immunohistochemistry. Data were reported as median and range. Paired non parametric Wilcoxon’s test and ANOVA of Kruskal-Wallis were used. The correlation between the contrast signal and the VEGFR-2 expression was assessed by the Spearman coefficient.

**Results:**

The Video intensity difference (VI_D_) caused by backscatter of the retained UCA_VEGFR-2_ was significantly higher in mice harboring thyroid tumors compared to mice with normal thyroids (*P* < 0.01) and to mice harboring benign nodules (*P* < 0.01). No statistically significant differences of VI_D_ were observed in the group of mice carrying benign nodules compared to mice with normal thyroids. Moreover in thyroid tumors VI_D_ of retained VEGFR-2-targeted UCA was significantly higher than that of control UCA_IgG_ (*P* <0.05). Results of immunohistochemical analysis confirmed VEGFR-2 overexpression. The magnitude of the molecular ultrasonographic signal from a VEGFR-2-targeted UCA retained by tissue correlates with VEGFR-2 expression determined by immunohistochemistry (*rho* 0.793, *P*=0.0003).

**Conclusions:**

We demonstrated that CEUS with UCA_VEGFR-2_ might be used for *in vivo* non invasive detection and quantification of VEGFR-2 expression in thyroid cancer in mice, and to differentiate benign from malignant thyroid nodules.

## Background

Angiogenesis is a critical determinant of tumor growth and invasion [1,2] and successful application of novel therapies that target tumor vasculature will require selection of susceptible tumors and precise evaluation of early treatment response. Vascular endothelial growth factor and its main receptor vascular endothelial growth factor receptor 2 (VEGFR-2), are overexpressed on tumor vascular endothelial cells and have been identified as targets for antiangiogenic drugs [3-10].

Papillary thyroid carcinoma (PTC) is the most common malignancy of the thyroid gland. At the molecular level PTC is characterized by genetic alterations of components of the mitogen-activated protein kinase (MAPK) pathway [11]. These include structural chromosome rearrangements affecting NTRK1 (TRK-T1) tyrosine kinase receptor that undergo in-frame recombination with various partner genes [12]. Specifically, the TRK-T1 oncogene results from a paracentric inversion of chromosome 1q25 that fuses the 5′ end of the TPR (Translocated Promoter Region) to the 3′ end of NTRK1 genes generating the constitutively active and oncogenic kinase NTRK1 [12]. Transgenic mice featuring the thyroid-specific expression of TRK-T1 under the transcriptional control of the thyroid-specific bovine thyroglobulin (Tg) promoter were generated previously [13]. Twenty-three% of TRK-T1 mice of age ≤ 7 months and 78% of mice > 7 months developed thyroid nodules characterized by malignant features, such as the proliferation of follicular epithelial cells containing scant cytoplasm, mitotic figures and papillae with fibrovascular stalks [13,14].

In papillary thyroid carcinoma, increased VEGFR-2 expression correlates with an increased cancer cell proliferation assessed by Ki-67 index, with increased thyroid tumor size [15,16] and with poor prognosis [16-19]. The thyroid cancer cells of primary tumors taken from patients with metastases had an higher VEGFR-2 expression compared to cells taken from primary tumors of patients without metastases [15,16]. These observations have been suggested to be clinically useful in identifying patients who are more prone to develop metastases.

Recently, tumor angiogenesis imaging *in vivo* has been noninvasively explored using contrast enhanced ultrasound (CEUS) with microbubbles (MBs) targeted to α_v_β_3_ integrin, endoglin, and VEGFR2 [20-24]. This technique is rapidly emerging as a noninvasive and quantitative molecular imaging modality that combines the advantages of high spatial resolution, real-time imaging, and lack of ionizing radiation and may be particularly advantageous in clinical oncology because VEGFR-2 has been implicated as marker of metastatic potential and poor prognosis in certain tumors [25-27].

Microbubbles are gas-filled echogenic US contrast agents that can be targeted to specific molecular markers by means of the attachment of appropriate ligands to the surface of the MBs. A specific characteristic of MBs is their relatively large size, which prevents them from leaking into the extravascular space. This property can be exploited for imaging by targeting the MBs to disease processes reflected on the vascular endothelial cells lining the luminal surface of capillaries and vessels, such as inflammation and angiogenesis. When these functionalized MBs are injected intravenously, they distribute throughout the whole body and attach at tissue sites expressing the targeted molecular marker, leading to a local increase of the US imaging signal. This approach allows the exclusive visualization of molecular markers of angiogenesis expressed on tumor vascular endothelial cells, have a potential clinical translation in future and should improve the ability to detect, diagnose stage, select appropriate treatments, and determine prognosis in patients with thyroid pathologies.

To our knowledge, no study has addressed the potential of targeted CEUS imaging for assessment of thyroid tumor angiogenesis *in vivo* by using MBs targeted to VEGFR-2.

This study aimed to investigate whether targeted CEUS allows noninvasive assessment of VEGFR-2 expression on tumor vascular endothelium in Tg-TRK-T1 mice, a murine model of thyroid cancer. We also investigated whether the evaluation of expression levels of VEGFR2 *in vivo* can differentiate benign from malignant nodules of the thyroid.

## Methods

### Animal model

Animal studies were performed in accordance with National Institutes of Health (NIH) recommendations and Animal Research Advisory Committee (ARAC) procedure [27] and the approval of the Italian Institutional animal research committee (Institutional Animal and Care Committee of the University of Naples “Federico II” and the Italian Ministry of Health). All animal procedures in this study were conducted by a veterinarian and conformed to all regulations protecting animals used for research purposes, including national guidelines [D.L. 27 Gennaio 1992, 116 Suppl. G.U 40 18 Febbraio 1992. Direttiva CEE n.609/86] as well as the protocols recommended by Workamn et al. [28].

Tg-TRK-T1 transgenic mice have been previously described [13]. From 2010 to 2011, thyroid Ultrasound was performed in 16 Tg-TRK-T1 transgenic mice model of thyroid carcinogenesis [26]. Body weight range of animals was 29–32 gr, equally distributed among male (n=9) and female (n=7). Mice were examined every six months and were sacrificed immediately after the last ultrasound scanning. At the time of the necroscopy, the age range of mice was 12–15 months.

### High frequency ultrasound with targeted contrast enhanced imaging

A Vevo 770 microimaging system (Visualsonics, Toronto, Ontario, Canada) with a single element probe, center frequency of 40-MHz was used for all the examinations. The transducer has an active face of 3 mm, a lateral resolution of 68.2 μm, axial resolution of 38.5 μm, focal length of 6 mm, mechanical index 0.14, transmit power 50%, and a dynamic range 52 dB [29,30]. Precise and repeatable control over the position of the two-dimensional image plane was obtained with a rail system (Vevo Integrated Rail System II; Visualsonics). Mice were anesthetized using 1.5–2% isoflurane vaporized in oxygen (2Lt/min) on a heated stage, with constant monitoring of their body temperature, using physiological monitoring platform [31]. Hairs were removed from the area of interest (neck and the high thorax) with a depilatory cream to obtain a direct contact of the ultrasound gel to the skin of the animal minimizing ultrasound attenuation. A prewarmed gel was used to provide a coupling medium for the transducer. Real-time imaging was performed as previously described [32]. The transducer focal zone was placed at the center of the thyroid gland and nodules, when they were present. All nodules were measured in three planes, and images were recorded to document nodule location and orientation. Each examination lasted for about 30 min. All ultrasonographic assessments were performed by the same trained sonographer (A.G.) that was not aware of the tumor type and of type of MBs administered to mice.

#### Contrast-enhanced agent preparation and injection

The Ultrasound Contrast Agent (UCA) MicroMarker (VisualSonics, Inc, Toronto, Ontario, Canada), specifically designed for high frequency ultrasonography, was prepared and targeted according to manufacturer guidelines. These MBs have a mean diameter of 1.5 μm (range, 1–2 μm) and contain approximately 7600 molecules of streptavidin per square micrometer chemically attached to the phospholipid shell of the MBs via a polyethylene glycol spacer [22]. The contrast agent preparation protocol was designed to achieve optimal saturation of the microbubble surface with a maximal amount of antibodies while minimizing the amount of free non conjugated antibodies in the solution. A vial of the dry UCA containing 9.2 × 10^8^ dry streptavidin-coated MBs was re-suspended in 1.2 mL of sterile saline. Then, either 30 μg of biotinylated anti-mouse VEGFR-2 antibodies (clone Avas12a1; eBioscience, San Diego,) or a biotinylated immunoglobulin G (IgG) isotype control (eBioscience, Inc, San Diego, CA) were added per vial of contrast agent to produce either a VEGFR-2-targeted (UCA_VEGFR-2_) and a control UCA (*UCA*_*IgG*_) by using biotin-streptavidin interactions, resulting in approximately 6000 ligands per square micrometer of surface area [22]. All mice were injected with two boluses of both the UCA_VEGFR-2_ and *UCA*_*IgG*_ via a tail vein (injection time, 2 seconds). Each bolus containing 3.8 × 10^7^ MBs in 0.02 mL of saline and was followed by a 0.02 mL saline flush.

To allow MBs to clear from previous injections, we waited at least 30 minutes between different bolus injections. The sequence of injections was always the same in all animals examined. The total amount injected was 80 μl.

#### Image acquisition and quantification

**Figure 1:**
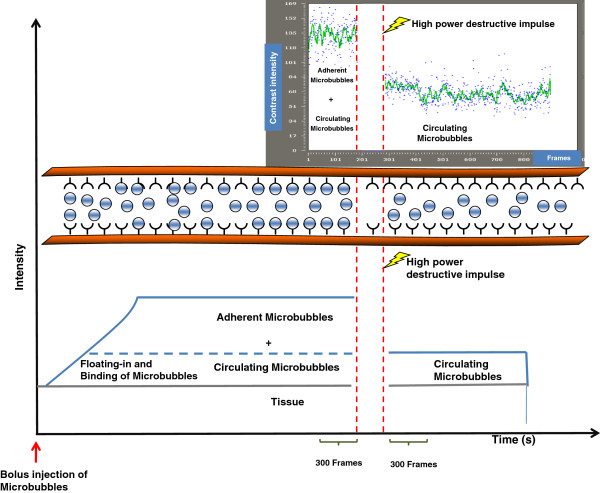
**Targeted US of endothelial antigens in vessels of a tumor tissue.** Endothelial cells of vessels (red) of tumor tissues expresses specific antigens. After intravenous administration targeted microbubbles (blue) float in vessels and remaining exclusively in the vascular compartment. Many of them bind to antigens of endothelial cells, whereas others remains in the vessel lumen freely circulating. After high-power destructive pulse, all microbubbles are destroyed (bound + circulating), following circulating microbubbles, that arrives from outside of scan plane, remain freely circulating for several seconds. On the top of the figure time/video intensity curve analysis before and after high-power destructive pulse and bottom a diagram representation of destructive methodology. Contrast intensity is the sum of the intensity from tissue, intensity from microbubbles not bound to receptors (circulating microbubbles), and intensity from microbubbles bound to receptors on endothelial cells. After digital subtraction of 300 predestruction frames from 300 postdestruction frames, resulting video intensity is due only to bound microbubbles.

The system was set at 50% transmit power, resulting in a mechanical index of 0.14 (manufacturer specification). Images were acquired at a 20-Hz frame rate. The data were log compressed and digitized to 12 bits. Data were further compressed to 8 bits for screen display. The ultrasound probe was positioned so that the central portion of the thyroid nodule was contained within the focal zone of the ultrasound transducer. The probe position, gain settings, and midfield focus were initially optimized and maintained throughout each experiment. The goal of the ultrasonographic image acquisition and analysis protocol (Figure 1) was to differentiate the backscattered acoustic signal due to MBs retained by the tumor from the background signal of the tumor itself and MBs still freely circulating in the bloodstream. CEUS imaging was paused for 4 minutes after injection. This time allowed binding and retention of targeted MBs while awaiting wash-out of the unbound contrast agent.

After the 4-minute waiting period, approximately 300 ultrasonographic frames of the tumor were acquired at a temporal resolution of 10 seconds. A high-power ultrasound destruction sequence was then applied (20 cycles with a frequency of 10 MHz and a mechanical index of 0.59). After the destruction pulse, the system was reset with identical imaging parameters as before the destruction event, and another set of images (≈300 frames) was acquired. Image processing and quantification were performed with the software implemented in the ultrasound scanner. Image processing used in the Vevo770 system relies on 2 sets of images: a predestruction set and a postdestruction data set. The received log compressed signals were expressed in an arbitrary scale unit called Video Intensity (VI). The average VI of predestruction and postdestruction (background) sonograms was measured in a region of interest encompassing the centre of examined tumor. The difference in VI between predestruction and postdestruction ultrasonographic frames was calculated and expressed as VI difference (VI_D_) that provided a relative measure of the amount of the UCA retained by the tumor and was considered to represent MBs adherent to molecular endothelial markers.

#### Histology and immunohistochemistry

After CEUS imaging, mice were euthanized, the thyroids were excised and immediately fixed in buffered formalin for 4 h. Tissues underwent automated processing and paraffin embedding; 5 μm sections were cut and hematoxylin and eosin stained for microscopic analysis. Thyroid tissues were classified according to the World Health Organization criteria for the evaluation of mouse thyroid tumors [33]. Briefly, thyroid was considered as normal when composed by variable-sized follicles covered by flattened monolayered epithelial cells. Hyperplastic thyroid was defined by the occurrence of small follicles with scant colloid and tall epithelial cells merging with normal areas. Follicular adenoma was defined as a well demarcated nodule with a distinct papillary and/or follicular architecture. Malignant lesions were defined based on the invasion of the surrounding glandular parenchyma and stroma.

To confirm expression of VEGFR-2, immunohistochemistry analysis of tumor sections was performed. Formalin-fixed and paraffin-embedded 3–5 μm sections were deparaffinized, placed in a solution of absolute methanol and 0.3% hydrogen peroxide for 30 min, and treated with blocking serum for 20 min. After blocking, slides were incubated with a mouse monoclonal anti-VEGFR-2 antibody (dilution 1:200) in a moist chamber at 4°C and processed according to standard procedures. Negative controls by omitting the primary antibody were included. Cases were scored as positive when unequivocal brown staining was observed. Immunoreactivity was expressed as the average percentage of positively stained target cells [(−): no staining (< 5% positive cells); (+): low/weak (≥ 5% - ≤ 25% positive cells); (++): medium/moderate (> 25% - < 50% positive cells); (+++): high/ strong (≥ 50% positive cells)]. Score values were independently assigned by two blinded investigators (G.C. and R.P.) and a consensus was reached on all scores used for computation. All histological and immunohistochemistry studies were performed and interpreted by pathologists, who did not know the diagnosis determined by ultrasonography.

#### Statistical analysis

Data were reported as median and range. Paired non parametric Wilcoxon’s test was used to compare data from different VI_D_ (*UCA*_*IGg*_ UCA_VEGFR-2_). ANOVA of Kruskal-Wallis was used to compare the contrast measurements of the three groups. *Post hoc* analysis was performed using the Dunn test. The correlation between the contrast signal and the VEGFR-2 expression was assessed by the Spearman coefficient.

A *P* < 0.05 was considered statistically significant. All statistical analysis were performed with MedCalc 12.0 statistical software.

## Results and discussion

Examination of the thyroid gland was performed by CEUS with *UCA*_*VEGFR-2*_ and *UCA*_*IgG*_. The UCA administration showed no notable toxicity, and all animals recovered after US imaging without any detectable signs of distress.

At the ultrasound evaluation in 16 mice examined: in 4 the thyroid showed normal size and homogeneous echotexture of parenchyma, without nodules, and therefore classified as normal, 2 mice showed features of benign diffuse hyperplasia and 10 mice had a nodular process.

At the histological examination 4 normal thyroids, 2 hyperplasias, 3 adenomas, 7 papillary thyroid carcinoma were found, confirming Ultrasound diagnosis. The benign thyroid nodules measured 0.11–0.27 mm (median 0.17 mm) while the tumors measured 0.16–5.51 mm (median 0.54 mm).

**Figure 2:**
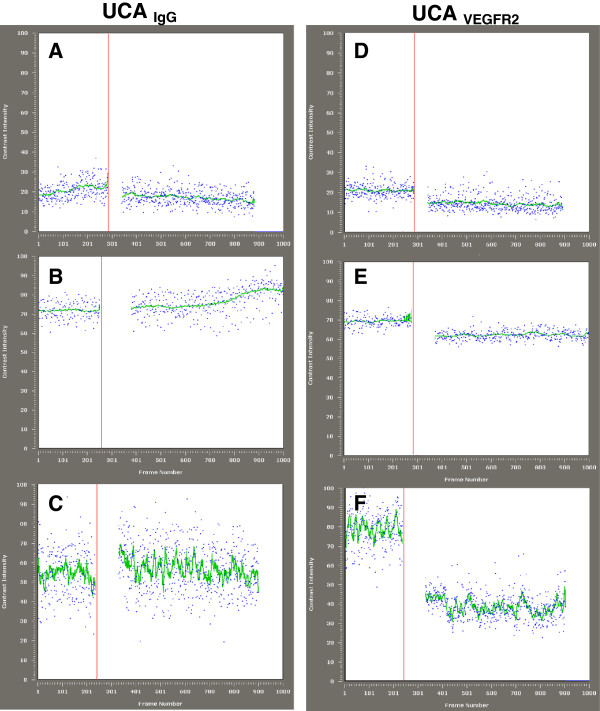
**Video intensities curves.** Predestruction and postdestruction video intensities curves for the control UCA **(A**–**C)** and the VEGFR2-targeted UCA **(D**–**F)**. The average video intensity of predestruction and postdestruction sonograms was measured and the difference in video intensity between the predestruction and postdestruction ultrasonographic frames was calculated and expressed as video intensity difference (VI_D_). This value provided a relative measure of the amount of targeted microbubbles retained by the tumor. Video Intensities curves of a normal thyroid parenchyma **(A**,**D)**, adenoma **(B**,**E)** and a thyroid tumor **(C**,**F)**. These images show a significant difference between retention of the control and VEGFR2-targeted UCAs in a thyroid tumor.

For thyroid tumors the VI_D_ was significantly higher when using *UCA*_*VEGFR-2*_ compared with *UCA*_*IgG*_ whereas for normal thyroids or in mice harboring benign thyroid nodules the VI_D_ for *UCA*_*VEGFR-2*_ was equal to the VI_D_ for *UCA*_*IgG*_ (Table 1 and Figure 2). Median range VI_D_ UCA_VEGFR2_ thyroid tumors, 30.1 (range 25.1-35.6) versus VI_D_*UCA*_*IgG*_ 19.4 (range 11.4-22.6) (*P*< 0.01).

**Table 1:** **Quantitative video intensity for ultrasound contrast agent targeted with isotype control antibody (UCA**_**IGg**_**) and anti-VEGFR2 monoclonal antibody (UCA**_**VEGFR2**_**)**

	**Normal thyroid (n.4)**	**Hyperplasia/Benign nodules (n.5)**	**Thyroid carcinoma (n.7)**
Video intensity UCA_IGg_			
Video intensity difference	11.3 (9.4-14.7)	12.2 (8.5-19.6)	19.4 (11.4-22.6)
Video intensity UCA_VEGFR2_			
Video intensity difference	10.9 (10.3-14.9)	13.3 (10.8-15.8)	30.1 (25.1-35.6)* §

* Statistical significant difference between Thyroid carcinoma and normal thyroid or Hyperplasia/benign nodules p<0.05.

§ Statistical significant difference between UCA targeted with anti-VEGFR2 monoclonal antibody and isotype control antibody p=0.0156.

Benign nodules VI_D_ UCA_*VEGFR-2*_ 13.3 (range 10.8-15.8) versus VI_D_*UCA*_*IgG*_ 11.82 (range 8.5-19.6) (*P*= n.s). Normal thyroids VI_D_ UCA_*VEGFR-2*_ 10.9 (range 10.3-14.9) versus VI_D_ in *UCA*_*IgG*_ 11.3 (9.4-14.7); (*P*= n.s) (Table 1). These values were used as relative measures of the VEGFR-2 over-expression within tumor vasculature and the *UCA*_*IgG*_ served as a control for demonstration of the specificity of *UCA*_*VEGFR-2*_ retention.

The median difference between VI_D_*UCA*_*IGg*_ and VI_D_ of UCA_VEGFR-2_ was considered as a measure of VEGFR-2 specific binding, was 11.6 (range 9.6-19.2) VI units for thyroid carcinoma significantly higher (p=0.0037) than for normal thyroid (median 0.3, range −1.57-1.32) and hyperplasia/benign nodules (median 2.3 range −3.8-3.0).

Figure 2 shows representative VI curves of a thyroid malignant nodule, normal thyroid and benign nodule imaged with the *UCA*_*VEGFR-2*_ and *UCA*_*IgG*_. There was a moderately intense signal from the *UCA*_*VEGFR-2*_ retained by the tumor (Figure 2F). The corresponding images for the *UCA*_*IgG*_ showed no retention of MBs in the tumor (Figure 2C). The *UCA*_*VEGFR-2*_ in the vascular bed of benign nodules and normal thyroids showed very low retention in *UCA*_*VEGFR-2*_ (Figure 2D-E).

After CEUS imaging, mice were subjected to general anesthesia and than euthanized.

In the group of examined animals the greatest *UCA*_*VEGFR-2*_ Video Intensity for normal or benign thyroid nodules was 15.8 units while the lowest *UCA*_*VEGFR-2*_ Video Intensity for carcinomas was 25.1 units. Therefore, we propose a cut-off value of 20 VI units to discriminate normal or benign nodules from malignant thyroid, that may be verified using a larger number of subjects.

**Figure 3:**
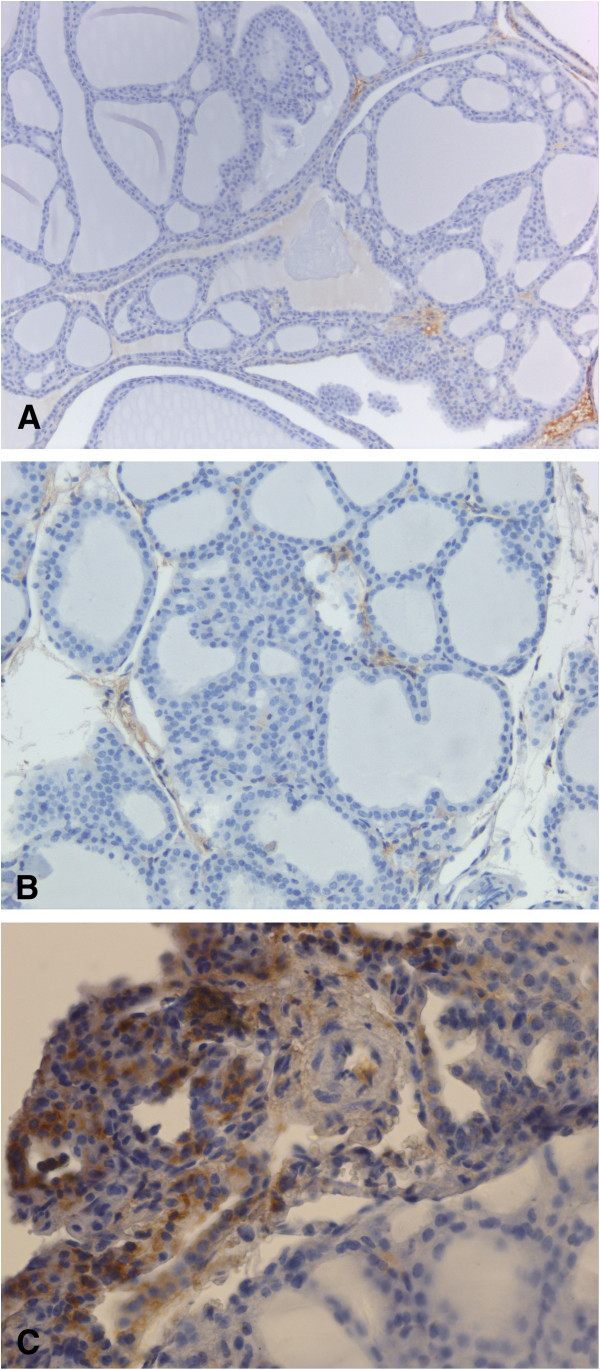
**Representative microphotographs of immunohistochemistry analysis of murine thyroid stained with antibodies against VEGFR type 2 receptor.** Brown color indicate presence of VEGFR2. Low grade expression of VEGFR-2 in normal thyroid **(A)**, in thyroid adenoma **(B)** and high grade expression in thyroid carcinoma **(C)**.

**Figure 4:**
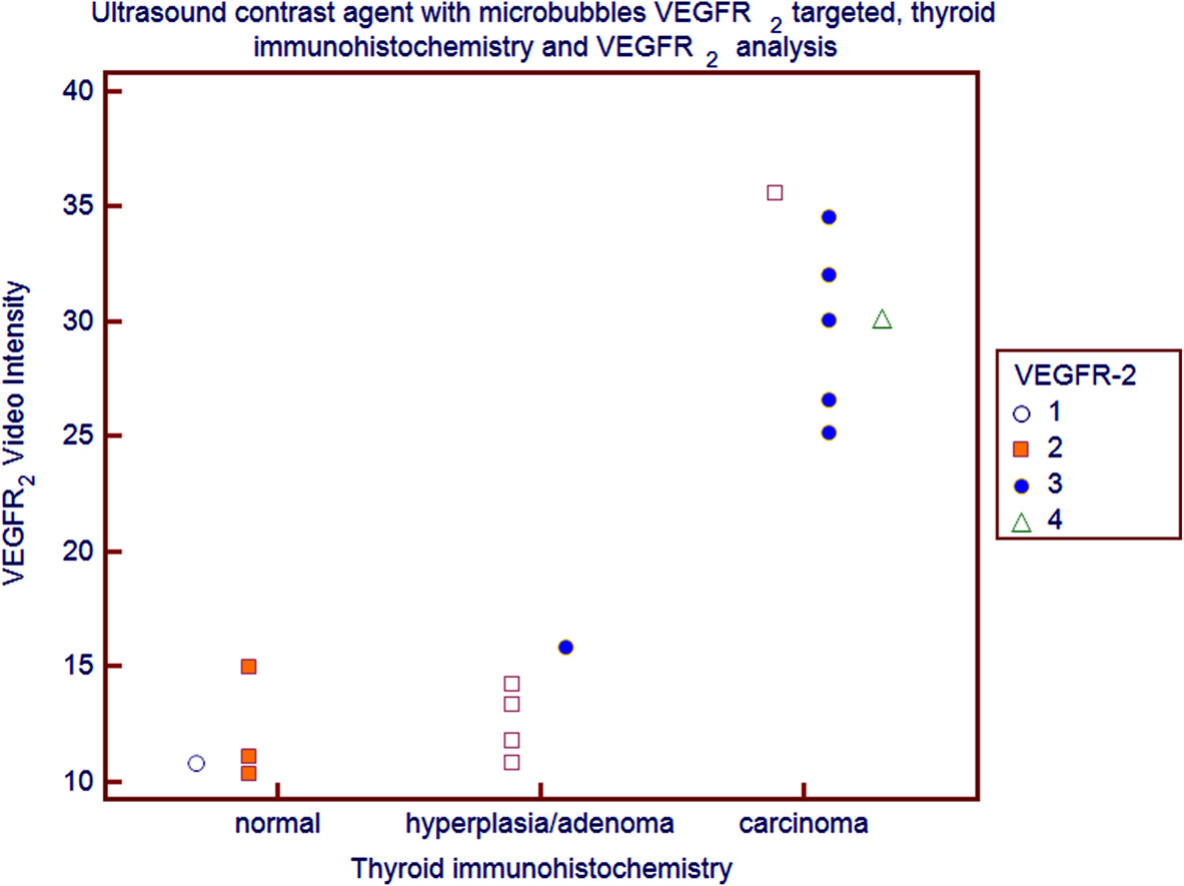
**Video Intensity Difference of VEGFR-2 targeted microbubbles and expression of VEGFR-2 determined by Immunohistochemistry expressed as the average percentage of positively stained cells in normal thyroids, in hyperplasia/adenoma and in carcinomas [white dot: no staining (< 5% positive cells); orange square: low/weak (≥ 5% - ≤ 25% positive cells); blue dot: medium/moderate (> 25% - < 50% positive cells); triangle: high/ strong (≥ 50% positive cells)].** The correlation was assessed by the Spearman coefficient.

To confirm expression of VEGFR-2, immunohistochemistry analysis of tumor sections was performed (Figure 3). The strength of the ultrasound signal from the *UCA*_*VEGFR-2*_ was significantly correlated with the level of actual VEGFR-2 expression (*rho* 0.793, *P*=0.0003) (Figure 4).

In this study we have evaluated the expression levels of a well-described tumor angiogenic marker i.e. VEGFR-2 in a mouse model of thyroid tumor (Tg-TRK-T1) compared with normal or benign tumors and whether targeted CEUS allows assessment of this marker noninvasively. The *in vivo* binding of the VEGFR-2 targeted UCA in thyroid tumors was substantially higher compared with control UCA. This difference in retention affirmed the specificity of a VEGFR-2- conjugated UCA for endothelial targeting. The UCA-IgG was higher in the tumors than in the benign thyroid nodules and normal thyroids, however the difference was not significant. Therefore targeting with VEGFR2 was necessary for differentiating a malignant tumor from a benign nodule. Vascular endothelial growth factor receptor 2 (VEGFR-2) is one of the best-characterized molecular marker of tumor angiogenesis [34-37]. It is overexpressed on tumor vascular endothelial cells in several solid tumors, including breast [38,39], ovarian [40,41], pancreatic [42] and thyroid cancer [19] and it is considered an important factor in tumor angiogenesis. VEGFR-2 is an endothelium-specific receptor tyrosine kinase that is activated by VEGF A. Activation of the VEGF/VEGFR-2 pathway triggers multiple signaling networks that result in endothelial cell survival, mitogenesis, migration, differentiation, and vascular permeability [43]. Insights into the expression levels of tumor angiogenic markers during the progression of cancer, could be of great importance in developing novel molecular imaging strategies aimed at visualization of tumor angiogenesis markers that are overexpressed in particular in early stage cancer for screening purposes.

The *in vivo* US imaging signals of the injected targeted UCA was correlated with results from immunochemistry analysis of VEGFR-2 expression and this positive correlation suggested that targeted contrast-enhanced US imaging could be used to monitor expression levels of angiogenic markers noninvasively (Figure 4). Thus retention of a VEGFR-2-targeted UCA is a more specific as *in vivo* marker for the level of VEGFR-2 expression than for the quantification of tumor vascularity.

The ability to visualize and quantify tumor angiogenesis may allow screening and detecting cancer at an early stage and antiangiogenic treatment monitoring in patients [34].

Targeted-CEUS is a promising non invasive molecular imaging approach that allows *in vivo* assessment of molecular markers of tumor angiogenesis [44-48]. The number of attached MBs depends on various factors, including the extent of tumor vascularization, physical forces that translate the freely circulating contrast MBs to the vessel wall, and the affinity of the binding ligand to the molecular targets, as well as the expression level of the molecular targets on tumor vessels [47-52].

Our study has several limitations. Molecular imaging of VEGFR-2 expression was performed in developed tumors (0.16–5.51 mm in diameter) in which it is very likely that the receptor is expressed at more. Thus, the usefulness and accuracy of VEGFR-2-targeted UCA imaging at earlier stages of tumor development needs to be evaluated.

A 2-dimensional image acquisition method was used, and it is very difficult to know whether ultrasound scans perfectly correspond with the region subjected to histological examination. Studies carried out in 3D mode could ensure greater correspondence between quantitative ultrasonographic assessment of VEGFR-2 expression and results of immunochemical analysis.

The small animal Vevo770 US system for dedicated small-animal imaging used in our study for MBs detection operates on received signals that undergo log-compression prior to image display. Log-compressed gray scale image values referred as “Video Intensity” can produce inaccurate estimation of perfusion user and instrument-dependent.

## Conclusions

The results of our study suggests that targeted CEUS imaging allows a non-invasive assessment of VEGFR-2 expression levels in thyroid *in vivo*. The results provide further insights into the biology of angiogenesis in thyroid tumors and may help in defining promising imaging targets for both early cancer detection and antitumor therapies.

## Competing interests

The authors declared that they have no competing interests.

## Authors’ contributions

MM conception and design of the study, analysis and interpretation of data, drafting the manuscript supervision of research group. AG carried out ultrasound studies, conception and design of the study, analysis and interpretation of data, and drafting of the manuscript. GS animal models, analysis and interpretation of data, drafting of the manuscript. RL design of the study, statistical analysis, interpretation of data. GDM collection of data, animal models. EV ultrasound studies, analysis of data. GC collection of data, animal models. RP collection of data, animal models. AB conceived of the study, and participated in its design and coordination and helped to draft the manuscript. MS conceived of the study, and participated in its design and coordination and helped to draft the manuscript. All authors read and approved the final manuscript.

## Pre-publication history

The pre-publication history for this paper can be accessed here:

http://www.biomedcentral.com/1471-2342/13/31/prepub

## References

[B1] Klener P Angiogenesis as part of the tumor “ecosystem” and possibilities to influence it Klin Onkol 2010 23 1 14 20 20192069

[B2] Pandya NM Dhalla NS Santani DD Angiogenesis–a new target for future therapy Vascul Pharmacol 2006 44 5 265 274 10.1016/j.vph.2006.01.005 16545987

[B3] Sato Y Molecular diagnosis of tumor angiogenesis and anti-angiogenic cancer therapy Int J Clin Oncol 2003 8 4 200 206 10.1007/s10147-003-0342-8 12955574

[B4] Sitohy B Nagy JA Dvorak HF Anti-VEGF/VEGFR therapy for cancer: reassessing the target Cancer Res 2012 72 8 1909 1914 10.1158/0008-5472.CAN-11-3406 22508695 PMC3335750

[B5] Kojic KL Kojic SL Wiseman SM Differentiated thyroid cancers: a comprehensive review of novel targeted therapies Expert Rev Anticancer Ther 2012 12 3 345 357 10.1586/era.12.8 22369326

[B6] Bertolini F Marighetti P Martin-Padura I Mancuso P Hu-Lowe DD Shaked Y D’Onofrio A Anti-VEGF and beyond: shaping a new generation of anti-angiogenic therapies for cancer Drug Discov Today 2011 16 23–24 1052 1060 21875682 10.1016/j.drudis.2011.08.007

[B7] Turner HE Harris AL Melmed S Wass JA Angiogenesis in endocrine tumors Endocr Rev 2003 24 5 600 603 10.1210/er.2002-0008 14570746

[B8] Warram JM Sorace AG Saini R Umphrey HR Zinn KR Hoyt K A triple-targeted ultrasound contrast agent provides improved localization to tumor vasculature J Ultrasound Med 2011 30 921 931 21705725 10.7863/jum.2011.30.7.921 PMC3140433

[B9] Ramsden JD Buchanan MA Egginton S Watkinson JC Mautner V Eggo MC Complete inhibition of goiter in mice requires combined gene therapy modification of angiopoietin, vascular endothelial growth factor, and fibroblast growth factor signaling Endocrinology 2005 146 7 2895 2902 10.1210/en.2005-0168 15817662

[B10] Nagura S Katoh R Miyagi E Shibuya M Kawaoi A Expression of vascular endothelial growth factor (VEGF) and VEGF receptor-1 (Flt-1) in Graves disease possibly correlated with increased vascular density Hum Pathol 2001 32 1 10 17 10.1053/hupa.2001.21139 11172289

[B11] Nikiforov YE Nikiforova MN Molecular genetics and diagnosis of thyroid cancer Nat Rev Endocrinol 2011 7 10 569 580 10.1038/nrendo.2011.142 21878896

[B12] Greco A Miranda C Pierotti MA Rearrangements of NTRK1 gene in papillary thyroid carcinoma Molecular and cellular endocrinology 2010 321 44 49 10.1016/j.mce.2009.10.009 19883730

[B13] Russell JP Powell DJ Cunnane M Greco A Portella G Santoro M Fusco A Rothstein JL The TRK-T1 fusion protein induces neoplastic transformation of thyroid epithelium Oncogene 2000 19 5729 5735 10.1038/sj.onc.1203922 11126359

[B14] Kim CS Zhu X Lessons from mouse models of thyroid cancer Thyroid 2009 19 1317 1331 10.1089/thy.2009.1609 20001715 PMC2861953

[B15] Klein M Catargi B VEGF in physiological process and thyroid disease Ann Endocrinol 2007 68 6 438 448 10.1016/j.ando.2007.09.004 17991452

[B16] Góth MI Hubina E Raptis S Nagy GM Tóth BE Physiological and pathological angiogenesis in the endocrine system Microsc Res Tech 2003 60 1 98 106 10.1002/jemt.10248 12500266

[B17] Salajegheh A Smith RA Kasem K Gopalan V Nassiri MR William R Lam AK Single nucleotide polymorphisms and mRNA expression of VEGF-A in papillary thyroid carcinoma: potential markers for aggressive phenotypes Eur J Surg Oncol 2011 37 1 93 99 10.1016/j.ejso.2010.10.010 21093207

[B18] Turner HE Nagy Z Gatter KC Esiri MM Harris AL Wass JA Angiogenesis in pituitary adenomas and the normal pituitary gland J Clin Endocrinol Metab 2000 85 3 1159 1162 10.1210/jc.85.3.1159 10720055

[B19] Risau W Angiogenic growth factors Prog Growth Factor Res 1990 2 1 71 79 10.1016/0955-2235(90)90010-H 1716501

[B20] Ellegala DB Leong-Poi H Carpenter JE Kaul S Shaffrey ME Sklenar J Lindner JR Imaging tumor angiogenesis with contrast ultrasound and microbubbles targeted to alpha(v)beta3 Circulation 2003 108 336 341 10.1161/01.CIR.0000080326.15367.0C 12835208

[B21] Korpanty G Carbon JG Grayburn PA Fleming JB Brekken RA Monitoring response to anticancer therapy by targeting microbubbles to tumor vasculature Clin Cancer Res 2007 13 323 330 10.1158/1078-0432.CCR-06-1313 17200371

[B22] Willmann JK Paulmurugan R Chen K Gheysens O Rodriguez-Porcel M Lutz AM Chen IY Chen X Gambhir SS US imaging of tumor angiogenesis with microbubbles targeted to vascular endothelial growth factor receptor type 2 in mice Radiology 2008 2 508 518 18180339 10.1148/radiol.2462070536 PMC4157631

[B23] Lee DJ Lyshchik A Huamani J Hallahan DE Fleischer AC Relationship between retention of a vascular endothelial growth factor receptor 2 (VEGFR2)-targeted ultrasonographic contrast agent and the level ofVEGFR2 expression in an in vivo breast cancer model J Ultrasound Med 2008 27 6 855 866 18499845 10.7863/jum.2008.27.6.855

[B24] Delorme S Krix M Contrast-enhanced ultrasound for examining tumor biology Cancer Imaging 2006 6 148 152 10.1102/1470-7330.2006.0023 17015239 PMC1693765

[B25] Klasa-Mazurkiewicz D Jarząb M Milczek T Lipińska B Emerich J Clinical significance of VEGFR-2 and VEGFR-3 expression in ovarian cancer patients Pol J Pathol 2011 62 1 31 40 21574104

[B26] Büchler P Reber HA Büchler MW Friess H Hines OJ VEGF-RII influences the prognosis of pancreatic cancer Ann Surg 2002 236 6 738 749 10.1097/00000658-200212000-00006 12454512 PMC1422640

[B27] Office of Animal Care and Use (OACU) of the National Institutes of Health (NIH) Animal Research Advisory Committee (ARAC) http://oacu.od.nih.gov/ARAC/

[B28] Workman P Aboagye EO Balkwill F Balmain A Bruder G Chaplin DJ Double JA Everitt J Farningham DAH Glennie MJ Kelland LR Robinson V Stratford IJ Tozer GM Watson S Wedge SR Eccles SA An ad hoc committee of the National Cancer Research Institute. Guidelines for the welfare and use of animals in cancer research Br J Cancer 2010 102 1555 1577 10.1038/sj.bjc.6605642 20502460 PMC2883160

[B29] Zhou YQ Foster FS Qu DW Zhang M Harasiewicz KA Adamson SL Applications for multifrequency ultrasound biomicroscopy in mice from implantation to adulthood Physiol Genomics 2002 10 2 113 126 12181368 10.1152/physiolgenomics.00119.2001

[B30] Greco A Mancini M Gargiulo S Gramanzini M Claudio PP Brunetti A Salvatore M Ultrasound biomicroscopy in small animal research: applications in molecular and pre-clinical imaging Journal of Biomedicine and Biotechnology 2012 Article ID 519238 14 10.1155/2012/519238 PMC3202139 22163379

[B31] The Australian and New Zealand Council for the Care of Animals in Research and Teaching Ltd (ANZCCART) Australia: The University of Adelaide http://www.adelaide.edu.au/ANZCCART/publications/

[B32] Mancini M Vergara E Salvatore G Greco A Troncone G Affuso A Liuzzi R Salerno P Scotto di Santolo M Santoro M Brunetti A Salvatore M Morphological ultrasound micro-imaging of thyroid in living mice Endocrinology 2009 150 10 4810 4815 10.1210/en.2009-0417 19589864

[B33] Jokinen MP Botts S Turusob VS, Mohr U WHO International Agency for Researchon Cancer Pathology of tumours in laboratory animals: tumours of the mouse Vol 2 1994 2 Lyon, France: IARC Scientific Publication 565 594

[B34] Palmowski M Huppert J Ladewig G Hauff P Reinhardt M Mueller MM Woenne EC Jenne JW Maurer M Kauffmann GW Semmler W Kiessling F Molecular profiling of angiogenesis with targeted ultrasound imaging: early assessment of antiangiogenic therapy effects Mol Cancer Ther 2008 7 1 101 109 18202013 10.1158/1535-7163.MCT-07-0409

[B35] Hodivala-Dilke K Alphavbeta3 integrin and angiogenesis: a moody integrin in a changing environment Curr Opin Cell Biol 2008 20 5 514 519 10.1016/j.ceb.2008.06.007 18638550

[B36] Ferrara N Vascular endothelial growth factor: basic science and clinical progress Endocr Rev 2004 25 4 581 611 10.1210/er.2003-0027 15294883

[B37] ten Dijke P Goumans MJ Pardali E Endoglin in angiogenesis and vascular diseases Angiogenesis 2008 11 1 79 89 10.1007/s10456-008-9101-9 18283546

[B38] Sledge GW Jr Rugo HS Burstein HJ The role of angiogenesis inhibition in the treatment of breast cancer Clin Adv Hematol Oncol 2006 4 10 Suppl 21 1 10 17139244

[B39] Khosravi Shahi P Soria Lovelle A Pérez Manga G Tumoral angiogenesis and breast cancer Clin Transl Oncol 2009 11 3 138 142 10.1007/S12094-009-0329-7 19293050

[B40] Gómez-Raposo C Mendiola M Barriuso J Casado E Hardisson D Redondo A Angiogenesis and ovarian cancer Clin Transl Oncol 2009 11 9 564 571 10.1007/s12094-009-0406-y 19775995

[B41] Bednarek W Mazurek M Cwiklińska A Barczyński B Expression of selected angiogenesis markers and modulators in pre-, peri- and postmenopausal women with ovarian cancer Ginekol Pol 2009 80 2 93 98 19338204

[B42] Saif MW Primary pancreatic lymphomas JOP 2006 7 3 262 273 16685107

[B43] Hicklin DJ Ellis LM Role of the vascular endothelial growth factor pathway in tumor growth and angiogenesis J Clin Oncol 2005 23 5 1011 1027 15585754 10.1200/JCO.2005.06.081

[B44] Lindner JR Microbubbles in medical imaging: current applications and future directions Nat Rev Drug Discov 2004 3 6 527 532 10.1038/nrd1417 15173842

[B45] Willmann JK van Bruggen N Dinkelborg LM Gambhir SS Molecular imaging in drug development Nat Rev Drug Discov 2008 7 7 591 607 10.1038/nrd2290 18591980

[B46] Pysz MA Foygel K Rosenberg J Gambhir SS Schneider M Willmann JK Antiangiogenic cancer therapy: monitoring with molecular US and a clinically translatable contrast agent (BR55) Radiology 2010 256 2 519 527 10.1148/radiol.10091858 20515975 PMC2909432

[B47] Willmann JK Kimura RH Deshpande N Lutz AM Cochran JR Gambhir SS Targeted contrast-enhanced ultrasound imaging of tumor angiogenesis with contrast microbubbles conjugated to integrin-binding knottin peptides J Nucl Med 2010 51 3 433 440 10.2967/jnumed.109.068007 20150258 PMC4111897

[B48] Lindner JR Song J Xu F Klibanov AL Singbartl K Ley K Kaul S Noninvasive ultrasound imaging of inflammation using microbubbles targeted to activated leukocytes Circulation 2000 102 22 2745 2750 10.1161/01.CIR.102.22.2745 11094042

[B49] Sorace AG Saini R Mahoney M Hoyt K Molecular ultrasound imaging using a targeted contrast agent for assessing early tumor response to antiangiogenic therapy J Ultrasound Med 2012 31 10 1543 1550 23011617 10.7863/jum.2012.31.10.1543 PMC3464103

[B50] Willmann JK Cheng Z Davis C Targeted microbubbles for imaging tumor angiogenesis: assessment of whole-body biodistribution with dynamic micro-PET in mice Radiology 2008 249 212 219 10.1148/radiol.2491072050 18695212 PMC2657857

[B51] Klibanov AL Rasche PT Hughes MS Wojdyla JK Galen KP Wible JH Jr Brandenburger GH Detection of individual microbubbles of ultrasound contrast agents: imaging of free-floating and targeted bubbles Invest Radiol 2004 39 3 187 195 10.1097/01.rli.0000115926.96796.75 15076011

[B52] Lucidarme O Kono Y Corbeil J Choi SH Golmard JL Varner J Mattrey RF Angiogenesis: noninvasive quantitative assessment with contrast-enhanced functional US in murine model Radiology 2006 239 3 730 739 10.1148/radiol.2392040986 16714458

